# Multispectral Imaging Analysis of Skin Lesions in Patients with Neurofibromatosis Type 1

**DOI:** 10.3390/jcm12216746

**Published:** 2023-10-25

**Authors:** Emilija V. Plorina, Kristine Saulus, Ainars Rudzitis, Norbert Kiss, Márta Medvecz, Tatjana Linova, Dmitrijs Bliznuks, Alexey Lihachev, Ilze Lihacova

**Affiliations:** 1 Institute of Atomic Physics and Spectroscopy, University of Latvia, LV-1586 Riga, Latvia; kristine.saulus@lu.lv (K.S.); aleksejs.lihacovs@lu.lv (A.L.); ilze.lihacova@lu.lv (I.L.); 2 LTD Longenesis, Dzirnavu 41A-5, LV-1010 Riga, Latvia; 3 Pauls Stradins Clinical University Hospital, Pilsoņu 13, LV-1002 Riga, Latvia; ainars_rudzitis@hotmail.com; 4 Department of Dermatology, Venereology and Dermatooncology, Semmelweis University, Maria Str. 41, H-1085 Budapest, Hungary; kiss.norbert@med.semmelweis-univ.hu (N.K.); medvecz.marta@med.semmelweis-univ.hu (M.M.); 5 Dermatology Clinic, Health Center 4, Skanstes 50, LV-1013 Riga, Latvia; tatjana.linova@vc4.lv; 6 Institute of Smart Computing Technologies, Riga Technical University, Zunda Krastmala 10, LV-1658 Riga, Latvia; dmitrijs.bliznuks@rtu.lv

**Keywords:** multimodal skin imaging, non-invasive skin imaging, rare disease diagnostics, multispectral imaging, neurofibromatosis type 1

## Abstract

Neurofibromatosis type 1 (NF1) is a rare disease, affecting around 1 in 3500 individuals in the general population. The rarity of the disease contributes to the scarcity of the available diagnostic and therapeutic approaches. Multispectral imaging is a non-invasive imaging method that shows promise in the diagnosis of various skin diseases. The device utilized for the present study consisted of four sets of narrow-band LEDs, including 526 nm, 663 nm, and 964 nm for diffuse reflectance imaging and 405 nm LEDs, filtered through a 515 nm long-pass filter, for autofluorescence imaging. RGB images were captured using a CMOS camera inside of the device. This paper presents the results of this multispectral skin imaging approach to distinguish the lesions in patients with NF1 from other more common benign skin lesions. The results show that the method provides a potential novel approach to distinguish NF1 lesions from other benign skin lesions.

## 1. Introduction

Neurofibromatosis type 1 (NF1), historically known as von Recklinghausen’s disease, is an autosomal dominant genetic condition caused by mutations in the NF1 gene, with a prevalence of 1 in 3500 individuals in the general population. It results in a few to thousands of neurofibromas, which are benign tumorous growths along the nerves. In addition, a variety of other neoplasms may develop.

NF1 is a multisystemic disorder with a highly variable clinical presentation that can affect multiple organs simultaneously. This often complicates the possibility of determining early diagnosis. Due to this, special diagnostic criteria were developed to help in identifying this disease. A summary of the diagnostic criteria for NF1 is compiled in Table 1. To declare a clinical diagnosis two or more of the criteria must be met [1].

Various signs of NF1 only appear over time—often at the onset of puberty. As a result, it is not possible to establish a definitive diagnosis of NF1 in a child or adolescent who only has a few café-au-lait spots [2]. Patients with NF1 have an increased risk of gliomas [3], malignant peripheral nerve sheath tumors (MPNSTs) [4], seizures [5], breast cancer [6], and other potentially severe complications.

There are several characteristic cutaneous manifestations of NF1. However, not all are present for each patient, and some are rarer than others. The two most common cutaneous lesions of NF1 are café-au-lait spots and dermal neurofibromas. Café-au-lait spots are round or oval coffee-colored macules, which get their color from increased melanin pigment content. They often appear within the first year of life. Dermal neurofibromas are nodules that appear during childhood and increase in number over the lifetime [7,8]. Neurofibromas are benign tumors of peripheral nerve sheaths, consisting of neoplastic cells, which are formed due to the impaired proliferation and differentiation of neural crest cells (melanocytes, Schwann cells, and endoneurial fibroblasts). Most often, neurofibromas arise in the skin or soft tissues from small nerves and are called nodular neurofibromas [9]. Other characteristic findings are plexiform neurofibromas, axillary or inguinal freckles (Crowe’s sign), and increased base pigmentation [7,8]. Unlike dermal neurofibromas, plexiform neurofibromas arise from multiple nerve fibers and can infiltrate the surrounding soft tissue, localized along nerve trunks [2]. Individuals with NF1 also have increased melanocyte density in the skin. The density is further elevated in individuals with café-au-lait macules who have NF1, while other individuals with café-au-lait macules but without NF1 do not exhibit this elevation [10].

The diagnosis of NF1 is usually based on clinical findings. However, since symptoms appear over time and may not be specific enough, the illness may be misdiagnosed or missed entirely [11]. Molecular genetic testing is increasingly being used to confirm the diagnosis based on the detection of NF1 gene mutations. Nevertheless, molecular genetic testing methods are not routinely used because they are time-consuming and expensive, and not all mutations are known [7]. For the assessment of manifestations affecting the central nervous system and other internal organs, magnetic resonance imaging (MRI) is used, while MPNSTs can be recognized using positron emission tomography [12,13]. For ophthalmologic changes, infrared reflectance imaging or optical coherence tomography may be used [14,15]. High-resolution ultrasound and dermoscopy may be used for the examination of dermal and superficial plexiform neurofibromas [16,17,18].

While there is no treatment for NF1, if a proper diagnosis is obtained, the symptoms can be managed and the patient can be monitored in case more severe manifestations develop. Cutaneous or subcutaneous neurofibromas can be removed surgically, using laser ablation or electrocautery. Pharmaceutics may be used for the management of neurological and cardiological symptoms. Malignant neurofibromas and other NF1-related tumors may warrant surgical excision and/or chemotherapy [10]. However, the rarity and variability of the disease means that many patients remain without diagnosis for many years, or even generations [19].

Multispectral imaging is able to provide information about the distribution of chromophores and fluorophores present in the skin. Since chromophores, such as hemoglobin, melanin, and water, have known absorption spectra [20,21,22], by using specific wavelengths that are close to the absorption maxima of certain chromophores, it is possible to obtain information about the distribution of blood, melanin, and water in the skin lesion. Also, by inducing autofluorescence, it is possible to obtain information about the content of fluorophores in the skin. The advantages of multispectral imaging compared to other methods for diagnosing NF1, mentioned by Yingjoy Li et al. [23], are less expensive (using equipment with different wavelengths of light sources) and easier to use (no special training and skills are required) hardware. In addition to combining images of chromophore distributions, it is possible to obtain the parameters responsible for the proportion of skin chromophores in the lesion, which, in turn, provides a greater contrast and allows different lesions to be distinguished from each other.

A novel multispectral imaging device, used for autofluorescence and diffuse reflectance imaging, was previously investigated for the differentiation of pigmented skin lesions and showed high accuracy [24]. Therefore, it could potentially be used for the evaluation of skin pigmentations of different backgrounds, such as café-au-lait macules and ephelides, among others. Café-au-lait macules may be misdiagnosed as congenital melanocytic nevus, Becker nevus, nevus spilus, lentigo solaris, urticaria pigmentosa, postinflammatory hyperpigmentation, plexiform neurofibroma, segmental pigmentation disorder, mastocytoma, phytophotodermatitis, and other pigmentation disorders [25]. If a patient does not have any neurofibromas, or has only a few, they may be misdiagnosed as schwannomas, perineuriomas, dermatofibromas, dermatofibrosarcoma protuberans, superficial leiomyomas, neurotized melanocytic nevi, ganglioneuromas, plexiform fibrohistiocytic tumors, desmoplastic melanomas, or other disorders [18,26].

The aim in this study was to compare NF1-related lesions to common, benign, noninflammatory lesions that are often checked in dermatology clinics, which is how the comparison groups of non-NF1-related lesions were chosen. Here, we set out to utilize multispectral imaging for the assessment of skin lesions in patients with neurofibromatosis type 1, including café-au-lait macules and neurofibromas.

## 2. Materials and Methods

### 2.1. Patient Data

All genetically confirmed NF1 patients in Latvia are registered at the Rare Disease Coordination Center in Riga. All known patients were invited to participate in the study. Of the invited patients, five individuals, aged 19 to 66 years (see Table 2) with genetically confirmed diagnoses of NF1, agreed to participate in the study. Measurements were organized from March to September 2022. Before measurements, the patients were examined by an expert dermatologist at the Health Center 4 Dermatology Clinic, who determined the nature, locations, and morphology of the skin lesions. General information about the patients and their lesions can be found in Table 2. In total, 90 multispectral datasets (65 of neurofibromas and 25 of café-au-lait macules) were collected from patients with NF1 diagnoses.

Multispectral datasets of 166 benign skin lesions, from 83 non-NF1 patients aged 18 to 79, that could be confused with neurofibromas or café-au-lait macules were used for comparison and included the following: 16 dermatofibromas, 4 intradermal nevi, 17 lentigines solaris, and 129 junctional nevi. These data were collected in the Oncology Center of Latvia, Riga, and the Department of Dermatology, Venereology and Dermato-oncology, Semmelweis University, Hungary, under the supervision of dermato-oncologists.

The study was approved by the Opinion of the Life and Medical Sciences Research Ethics Committee of the University of Latvia (signed by L. Plakane in Latvia, 30 June 2021 and S. Mežinska in Latvia, 14 June 2022) and complies with EU data privacy laws. All included subjects provided their informed consent to participate in the study.

### 2.2. Imaging Setup

A multispectral imaging device developed in collaboration between University of Latvia and Riga Technical University in Riga, Latvia was used for the study. The utilized imaging device is encased in a 3D printed outer shell. It consists of four sets of narrow-band LEDs—526 nm, 663 nm, and 964 nm for diffuse reflectance imaging, and 405 nm LEDs, filtered through a 515 nm long-pass filter, for autofluorescence imaging—an IDS 5-megapixel CMOS camera (IDS uEye, Obersulm, Germany) and a Raspberry Pi module (Raspberry Pi, Pencoed, United Kingdom) for data processing, and a 4G cellular modem (Huawei, Dongguan, China) for data transmission to a cloud server. A linear polarizer (Edmund Optics, York, United Kingdom) was placed over the LEDs, as well as another one perpendicularly over the camera lens, to reduce the effects of direct reflections. As a result, one multispectral dataset contained 4 RGB images, which were further processed. The measurement of all four images takes up to approximately 10 s, depending on the quality of the 4G connection at the specific location. The structure and contents of the multispectral device are described further in a paper by Osipovs et al. [27].

### 2.3. Data Processing

Data pre- and postprocessing were conducted using MATLAB and Python programming languages. For diffuse reflectance imaging, the following channels from the RGB images were used to extract the signal: a G channel for images taken under 526 nm illumination, an R channel for images taken under 663 nm illumination, and an R channel for images taken under 964 nm illumination. For autofluorescence imaging, the G channel was extracted from the RGB images prior to further processing, as it contains the most informative part of the autofluorescence signal [28].

The images required stabilization before further processing. Prior to capturing the images, in order to improve the stabilization procedure following data acquisition, a black marker was placed on the patient’s skin close to the lesion. Stabilization was performed using an algorithm that was developed in conjunction with image parameter calculation. This algorithm finds the darkest pixels of the image to determine the location of the marker, and the images are then superimposed so that the marker on each image matches.

These steps were followed by image segmentation, which was performed using the thresholding segmentation method. As described previously, the marker is segmented by finding the darkest pixels of the image. Since the marker is located on healthy skin next to the lesion, it can be further used to calculate the average range of pixel values of the perilesional skin. The marker segment is dilated so that it contains skin, and then it is subtracted from the image. In the following step, the segment of the lesion can be found if the skin and marker pixel values are removed from the image.

This method creates sufficiently precise segments in around 80% of cases, especially if the lesion pixel values are very different from the skin and marker values, and the lesion area has pronounced edges, which is useful when processing large batches of data. For the remaining percentage of the data, the segmentation masks were created or corrected using ImageJ v.1.53t software (NIH, Bethesda, MD, USA).

The same lesion area pixels can then be extracted from each multispectral image for further processing.

The pixels within the lesion area were extracted using the lesion segmentation mask from all four multispectral images. The parameters for each were then denoted as follows: AF(lesion) for autofluorescence intensity values of the lesion area; G(lesion) for the 526 nm or green-colored diffuse reflection values of the lesion area; R(lesion) for the 663 nm or red colored diffuse reflection values of the lesion area; and IR(lesion) for the 964 nm or infrared colored diffuse reflection values of the lesion area. From the skin segment, the pixel values of the skin area, AF(skin), G(skin), R(skin), and IR(skin), can be extracted.

G, R, and IR of the skin and lesion area values are used to calculate the parameter *p*′, which has previously been shown to be sensitive to the differentiation of malignant skin tumors from benign lesions [24].

The formula for this parameter is as follows:

(1) $$p^{\prime }={log}_{10}\left(\frac{G(lesion)\cdot R(skin)\cdot IR(skin)}{G(skin)\cdot R(lesion)\cdot IR(lesion)}\right);$$

From the calculated pixel values, statistical parameters were calculated, including the mean, variance, standard deviation, and mean of maximum and minimum 5% of the lesion pixel values.

**Figure 1:**
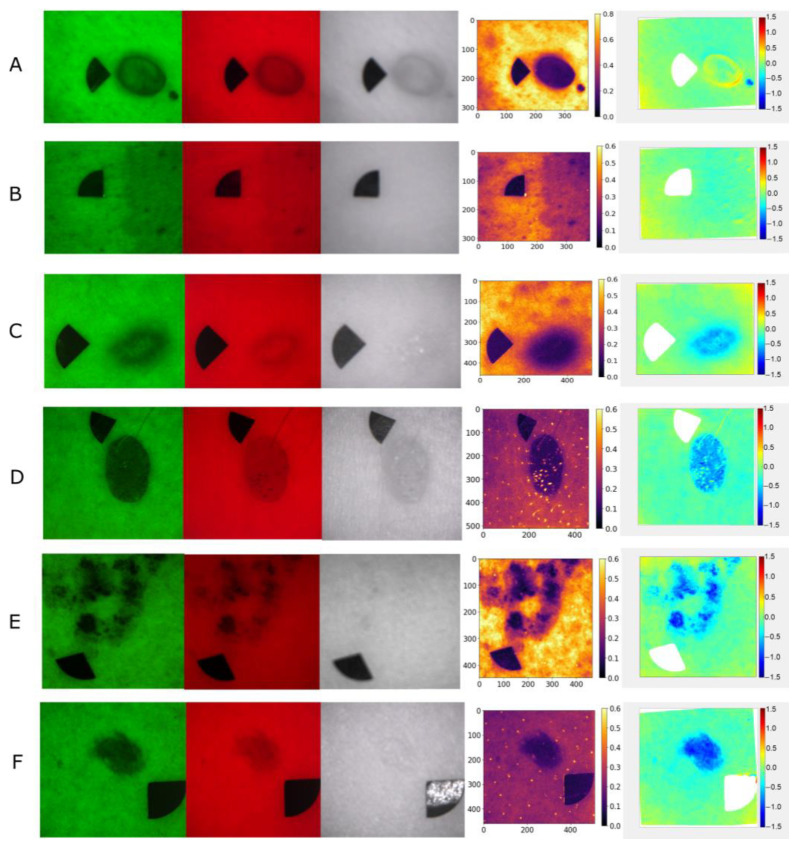
Multispectral images of a neurofibroma (**A**), a café-au-lait macule (**B**), a dermatofibroma (**C**), an intradermal nevus (**D**), a lentigo solaris (**E**), and a junctional nevus (**F**); from left to right: under 526 nm, 663 nm, 964 nm illumination, autofluorescence intensity in G channel, and calculated parameter *p*′.

Examples of raw multispectral images, autofluorescence intensity, and *p*′ maps of the lesion types used in the study are shown in Figure 1.

The sensitivity and specificity of parameters were calculated using the following formulas:

(2) $$Sensitivity=\frac{True\;Positives}{True\;Positives+False\;Negatives}\cdot 100\%;$$

(3) $$Specificity=\frac{True\;Negatives}{True\;Negatives+False\;Positives}\cdot 100\%;$$

## 3. Results

**Figure 2:**
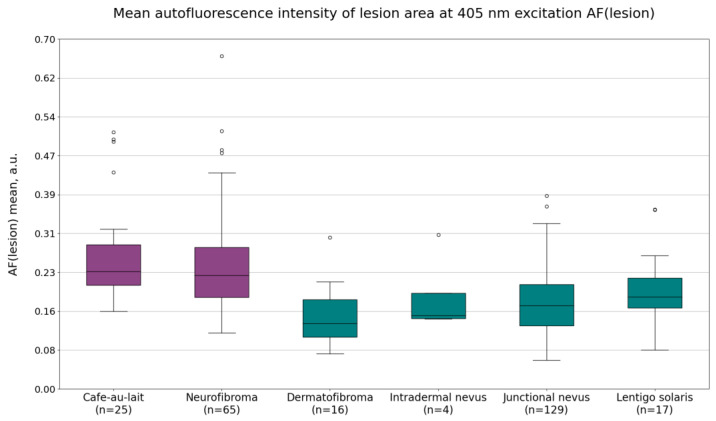
The calculated mean autofluorescence intensity values of café-au-lait macules, neurofibromas, dermatofibromas, intradermal nevi, junctional nevi, and lentigines solaris.

**Figure 3:**
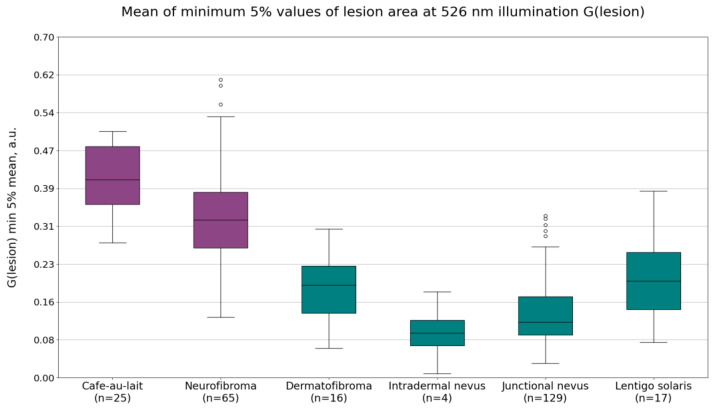
The calculated mean minimum 5% of values of the parameter G(lesion), or the lesion area pixel values of café-au-lait macules, neurofibromas, dermatofibromas, intradermal nevi, junctional nevi, and lentigines solaris under 526 nm illumination.

**Figure 4:**
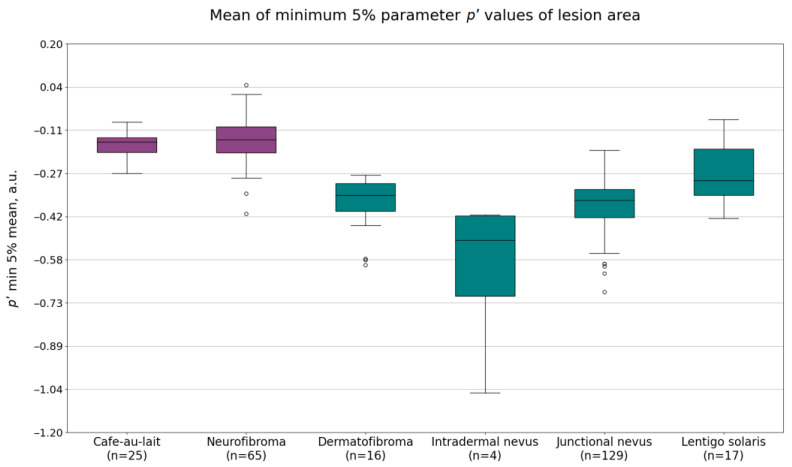
The calculated mean of minimum 5% values of the *p*′ parameter of the lesion area of café-au-lait macules, neurofibromas, dermatofibromas, intradermal nevi, junctional nevi, and lentigines solaris.

The imaging data from neurofibromas and café-au-lait macules were compared to the data from dermatofibromas, intradermal nevi, lentigines solaris, and junctional nevi. The calculated mean autofluorescence intensity values, mean minimum 5% of the values of the G(lesion) or lesion area pixel values under 526 nm illumination, and the calculated minimum 5% values of the *p*′ parameter of the lesion area are shown in Figure 2, Figure 3 and Figure 4. Diffuse reflectance imaging parameters R(lesion) and IR(lesion) did not show any significant differences. Therefore, these parameters were excluded from further analysis.

Mean autofluorescence intensity values AF(lesion) are shown in Figure 2. On average, the mean autofluorescence intensity was higher for NF1-related lesions (cafe-au-lait macules and neurofibromas) when compared to non-NF1-related lesions (dermatofibromas, intradermal nevi, junctional nevi, and lentigines solaris). The average values were as follows: 0.27 ± 0.10 a.u. for café-au-lait macules, 0.25 ± 0.11 a.u. for neurofibromas, 0.15 ± 0.11 a.u. for dermatofibromas, 0.19 ± 0.08 a.u. for intradermal nevi, 0.17 ± 0.07 a.u. for junctional nevi, and 0.20 ± 0.08 a.u. for lentigines solaris.

Diffuse reflectance intensity values under 526 nm illumination G(lesion) showed significant differences between café-au-lait macules and intradermal or junctional nevi, dermatofibromas, and lentigines solaris, but was less pronounced between neurofibromas and the other benign lesions. The most useful parameter was found to be the mean of minimum 5% of the pixel values (Figure 3). The average values of this parameter were 0.41 ± 0.07 a.u. for café-au-lait macules, 0.33 ± 0.10 a.u. for neurofibromas, 0.18 ± 0.10 a.u. for dermatofibromas, 0.09 ± 0.07 a.u. for intradermal nevi, 0.13 ± 0.07 a.u. for junctional nevi, and 0.20 ± 0.09 a.u. for lentigines solaris. NF1-related lesions, on average, have higher diffuse reflectance intensity values under 526 nm illumination than non-NF1-related lesions.

Finally, the parameter *p*′ also showed potential for distinguishing NF1 lesions from other benign lesions. The mean of minimum 5% of the pixel values showed a larger difference between NF1-related lesion values and non-NF1-related lesion parameter values compared to the mean value of the parameter. The average values of the mean of minimum 5% of the *p*′ values were −0.16 ± 0.05 a.u. for café-au-lait macules, −0.15 ± 0.08 a.u. for neurofibromas, −0.38 ± 0.11 a.u. for dermatofibromas, −0.57 ± 0.25 a.u. for intradermal nevi, −0.38 ± 0.09 a.u. for junctional nevi, and −0.25 ± 0.10 a.u. for lentigines solaris.

Table 3 summarizes specificity and sensitivity (95% confidence interval (CI)) results when comparing NF1 and non-NF1-related lesions using the following parameters: mean of G(lesion) means, mean of G(lesion) minimum 5% values, mean of *p*′, and mean of *p*′ minimum 5% values.

When comparing café-au-lait macules with non-NF1-related lesions, the mean of G(lesion) minimum 5% values had the highest accuracy, showing a sensitivity of 100% and a specificity of 95%. The parameter mean of *p*′ minimum 5% values had slightly lower values, with 96% sensitivity and 96% specificity. Intradermal nevi were distinguished from café-au-lait macules by all compared parameters with 100% specificity. Using the mean of *p*′ minimum 5% values, dermatofibromas were distinguished with 100% specificity, while junctional nevi were distinguished with 96% specificity. The hardest to distinguish were lentigines solaris, with the highest specificity value being 82% using mean of G(lesion) minimum 5% values.

When comparing neurofibromas with non-NF1-related lesions, the mean of *p*′ minimum 5% values showed the highest accuracy–sensitivity, with 94% sensitivity and 80% specificity. Using this parameter, intradermal nevi could be distinguished with 100% specificity, junctional nevi with 84% specificity, dermatofibromas with 88% specificity, and lentigines solaris with 35% specificity.

Overall, neurofibromas are more difficult to distinguish from the non-NF1-related lesions using the calculated parameters compared to café-au-lait macules. This affected the values calculated when comparing all NF1-related lesions and all non-NF1-related lesions. The parameter with the highest combination of sensitivity and specificity is the mean of *p*′ minimum 5% values, with 94% sensitivity and 84% specificity.

## 4. Discussion

The results obtained in this study indicate that the most informative parameter with which to distinguish NF1-related lesions from non-NF1-related lesions is the mean of minimum 5% of the *p*′ values. When comparing café-au-lait macules to non-NF1-related lesions, the most informative parameter is the mean of minimum 5% of the G(lesion) values. Since the NF1-related lesions are partially distinguishable using the parameter G(lesion), it could be assumed that the observation of parameter *p*′ having good accuracy comes from inclusion of G(lesion) in its formula. However, as shown in Table 3, in the case when only neurofibromas are compared, *p*′ still shows higher accuracy than only the parameter G(lesion). Parameter *p*′ includes G(lesion), R(lesion) and IR(lesion), as well as the diffuse reflectance values of the skin in the same images, G(skin), R(skin), and IR(skin). Since the influences of R(lesion) and IR(lesion) on the differentiation of lesions were not observed (therefore, further analysis of these channels was excluded), it could be concluded that the composition of the skin in each individual patient has a significant effect, and when comparing skin lesions between different patients, the diffuse reflectance values from the healthy skin of patients should also be taken into account (for example, the *p*′ parameter). Considering these parameters separately, the parameter *p*′ would be superior because it also includes the diffuse reflectance values of the healthy skin.

Light in the green wavelength range has been shown to be most sensitive in the visible spectrum to both oxy- and deoxy-hemoglobin in the skin [29]. However, the absorption is dependent on the amount of oxygen bound to the hemoglobin molecule [30]. The absorption maxima in the visible spectrum have been measured at 542 nm and 576 nm for oxy-hemoglobin and 556 nm for deoxy-hemoglobin [31]. A shorter-wavelength LED was used in the present study due to unavailability of an LED with a wavelength closer to the maximum; nevertheless, the imaged reflectance signal would still be significantly affected by the hemoglobin present in the skin. Therefore, the previously described parameter G(lesion) could indicate a difference in hemoglobin concentrations within the lesion area. Non-NF1-related lesions showed decreased signals under 526 nm LED illumination compared to NF1-related lesions. According to previous research, nevi have decreased blood vessel density values when compared to the surrounding skin [32], while neurofibromas’ blood vessel density is comparable to that of normal skin [20,33]. Therefore, if hemoglobin was in the background of a different reflectance signal under 526 nm illumination for certain neurofibromas and nevi, the difference would have been inverted—neurofibromas would have had, on average, a lower intensity due to higher absorbance. On the other hand, dermatofibromas have been shown to have a higher blood vessel density; hence, the lower intensity could be explained by increased hemoglobin [34]. Blood vessel density measurements of café-au-lait macules were not found in the literature, but their histological description [35] does not describe changes in circulation, which could mean that they have similar blood vessel density to normal skin, similarly to neurofibromas. Lentigines solaris show increased blood vessel density when compared to normal skin [36], which could mean that the differentiation of café-au-lait macules from them under 526 nm illumination is related to an increased hemoglobin concentration.

Since the relationship of parameter G(lesion) between nevi and NF1-related lesions was not inverted, it is highly likely that the epidermal and dermal melanin content, or the structure of the lesion that determines the distribution of melanin is more important for this parameter when NF1-related lesions are compared to intradermal and junctional nevi. While there are pigmented neurofibromas, most of them are not, which would decrease their absorption and increase reflectance intensity. However, café-au-lait macules are distinctly pigmented lesions. The results may be affected by how keratinocytes are distributed with melanin in these lesions—in café-au-lait macules, they are more spread out, while nevi are more clustered, which would also decrease overall absorption. In addition, nevi also have characteristically high numbers of melanocytes in them, while café-au-lait macules are composed mostly of keratinocytes [37,38].

The previously described relationships between chromophore content and reflectance parameters would apply to the parameter *p*′, as well. According to previous research, the parameter *p*′ was created by considering the absorption spectra of hemoglobin, melanin, and water [39].

While NF1-related lesions showed, on average, higher autofluorescence signal intensity values compared to non-NF1-related lesions, they could not be distinguished well when compared to the reflectance measurements. Autofluorescence is usually observed in the upper layers of the skin. These results suggest that the structural or fluorophore changes between these two lesion groups are not well observed using autofluorescence measurements.

The result that minimum 5% values showed a higher distinction than normal mean for both G(lesion) and parameter *p*′ was already anticipated during preprocessing, which is why these values were extracted in the first place. The image lesion masks created during the segmentation process are imperfect and may include skin pixels due to image stabilization issues and lack of sharp borders. Since skin is lighter than all the captured lesions in the images, the minimum values specifically would be closer to the values of the lesion, instead of those of the surrounding skin. This is also a limitation of the method because it relies on uniform exposure of the image, and these parameters may not produce results if an image is underexposed.

Other limitations of this study include the limited number of NF1 patients, as well as the lack of diversity in age and sex in these patients. Since the patients were not invited directly, but through the Rare Disease Coordination Center, it may be that patients in this age range and of this sex are more likely to participate in studies. To confirm the correlations, more images should be collected, and the calculations should be repeated. To obtain more data, it would be necessary to approach new, undiagnosed patients, as well as to expand the study beyond Latvia’s borders.

## 5. Conclusions

In this work, we have shown that there is a potential for distinguishing the skin lesions of NF1 from similar, more common, skin lesions—dermatofibromas, intradermal nevi, junctional nevi, and lentigines solaris—using a multispectral imaging approach. Images taken under the illumination of 526 nm LEDs showed the highest accuracy for the differentiation of café-au-lait macules from non-NF1-related lesions, with 95% specificity (100% sensitivity) when using the mean of minimum 5% of the intensity values. For neurofibromas, the highest degree of differentiation was shown with parameter *p*′, with 80% specificity (94% sensitivity). Overall, parameter *p*′ performed the best for distinguishing NF1-related lesions from lentigines solaris, dermatofibromas, intradermal nevi, and junctional nevi, with 84% specificity (94% sensitivity).

However, it should be considered that these are initial attempts to create multispectral imaging technology for the assessment of NF1 lesions, so the amount of data, in terms of the fact that the number of patients with rare diseases, is small, and the dispersion of the created parameters may be greater with a larger number of patients. Currently, this method requires additional data and confirmation before the implementation of this method in dermatologists’ or family doctors’ practices.

## Figures and Tables

**Table 1:** The diagnostic criteria of NF1 [1].

>6 café-au-lait macules (prepubertal >5 mm, postpubertal >15 mm in size)
Axillary or inguinal freckling
Two or more neurofibromas or one plexiform neurofibroma
Optic nerve glioma
Two or more iris hamartomas (Lisch nodules)
Distinctive osseous lesion (such as sphenoid wing dysplasia or thinning of long bone cortex with or without pseudoarthrosis)
Variant allele fraction of 50% in tissues, such as white blood cells with a heterozygous pathogenic NF1 variant
A primary relative with NF1 with the above criteria

**Table 2:** NF1 patient demographics and the characteristics of their skin lesions.

Pt. No.	Sex	Age (Yrs)	Localization and Type of Lesions
1	F	66	Neurofibromas localized on the chin and upper chest; no café-au-lait macules
2	F	48	Multiple neurofibromas across the body; no café-au-lait macules
3	F	52	Multiple neurofibromas across the body; café-au-lait macules present on body
4	M	19	No neurofibromas; multiple café-au-lait macules across the body
5	F	43	Multiple neurofibromas across the body; café-au-lait macules present on the body

**Table 3:** Summary of sensitivity and specificity values when comparing café-au-lait macules, neurofibromas, and all NF1-related lesion group parameter values to lentigo solaris, dermatofibromas, intradermal nevi, junctional nevi, and all non-NF1-related group parameter values, with a 95% confidence interval (highlighted cells indicate the highest values when comparing NF1-related lesions and all non-NF1-related lesions).

NF Related Lesion Group	Parameter	Sensitivity (CI 95%)	Specificity of Parameter with Comparison Group (CI 95%)				
			All Non-NF Related Lesion Groups	Lentigo Solaris	Dermato-fibroma	Intradermal Nevus	Junctional Nevus
Café-au-lait macules	Mean of G(lesion)	100%	81%	65%	88%	100%	82%
	Mean of G(lesion) minimum 5% values	100%	95%	82%	94%	100%	96%
	Mean of *p*′	96%	70%	35%	69%	100%	74%
	Mean of *p*′ minimum 5% values	96%	93%	65%	100%	100%	96%
Neurofibroma	Mean of G(lesion)	94%	30%	12%	25%	50%	33%
	Mean of G(lesion) minimum 5% values	94%	52%	24%	25%	75%	58%
	Mean of *p*′	95%	57%	18%	63%	75%	61%
	Mean of *p*′ minimum 5% values	94%	80%	35%	88%	100%	84%
All NF related lesions	Mean of G(lesion)	96%	41%	18%	44%	100%	42%
	Mean of G(lesion) minimum 5% values	94%	62%	41%	31%	75%	68%
	Mean of *p*′	97%	73%	35%	75%	100%	77%
	Mean of *p*′ minimum 5% values	94%	84%	53%	88%	100%	88%

## Data Availability

Data are available on request.
